# Enabling neighbour labelling: using synthetic biology to explore how cells influence their neighbours

**DOI:** 10.1242/dev.201955

**Published:** 2024-01-02

**Authors:** Mattias Malaguti, Tamina Lebek, Guillaume Blin, Sally Lowell

**Affiliations:** Centre for Regenerative Medicine, Institute for Stem Cell Research, School of Biological Sciences, University of Edinburgh, 5 Little France Drive, Edinburgh EH16 4UU, UK

**Keywords:** Cell-cell interactions, Image analysis, Neighbour labelling, SynNotch, Synthetic signalling

## Abstract

Cell-cell interactions are central to development, but exploring how a change in any given cell relates to changes in the neighbour of that cell can be technically challenging. Here, we review recent developments in synthetic biology and image analysis that are helping overcome this problem. We highlight the opportunities presented by these advances and discuss opportunities and limitations in applying them to developmental model systems.

## Teaser

**Summary:** This Primer reviews emerging approaches for engineering synthetic signalling between cells and discusses how these could be applied to study the influence cells have on their neighbours during development.

## Introduction

Developmental biology examines how complexity emerges through interactions between cells. To understand how this happens, it would be useful to be able to profile the molecular events that cells undergo in response to particular changes in their neighbours. However, in contrast to the plethora of well-established single-cell-omics approaches for profiling cell-autonomous events (Vandereyken et al., 2023), methods for unbiased ‘neighbourhood profiling’ have only recently begun to emerge (Bechtel et al., 2021).

Neighbourhood-profiling methods generally rely on synthetic re-engineering of native signalling pathways to apply or induce a fluorescent label within the neighbour of a particular cell type or cell state. These labelled neighbours can then be extracted and subjected to functional assays or omics analysis. Variations of this approach can be adapted for controlling (Garibyan et al., 2023 preprint; Malaguti et al., 2022; Morsut et al., 2016; Toda et al., 2018), as well as monitoring (Lebek et al., 2023 preprint; Ombrato et al., 2019; Talay et al., 2017; Zhang et al., 2022), local interactions between live cells. Some of these methods label only direct neighbours (Morsut et al., 2016), whereas others the local cellular neighbourhood (Lebek et al., 2023 preprint; Ombrato et al., 2019) (Box 1). In parallel, recent innovations in computational image analysis are making it ever more feasible to reliably interrogate neighbour relationships in 3D tissues or embryos (Gómez et al., 2021; Guirao et al., 2015; Heller et al., 2016; Hicks et al., 2019; Shah et al., 2019; Stadler et al., 2018; Strauss et al., 2022; Summers et al., 2022; Toth et al., 2018), providing a complementary approach for exploring how cells influence their neighbours and for validating candidates that emerge from neighbour-profiling experiments (Box 1).

**Box 1. Defining neighbours in image analysis**

In everyday life, do we use the word ‘neighbour’ to refer only to the people who live directly next door or would we consider our friend a few doors down the street to also be our neighbour? Similar questions arise when defining relationships between cells in microscopy images (Gómez et al., 2021; Guirao et al., 2015; Heller et al., 2016; Hicks et al., 2019; Shah et al., 2019; Stadler et al., 2018; Strauss et al., 2022; Summers et al., 2022; Toth et al., 2018) (Fig. 6). When performing image analysis, it becomes the decision of the experimenter to determine how they wish to define a ‘neighbour’, highlighting an important distinction between image-analysis approaches and synthetic neighbour-labelling approaches, where ‘neighbours’ are defined according to the particular design of the labelling system.

Recent advances in image analysis have greatly improved our ability to ask how the properties of particular cells relate to the properties of their neighbours. Improvements in cell segmentation now make it possible to quantify fluorescence levels in individual cells even within crowded 3D tissues, cultures or embryos (Berg et al., 2019; Blin et al., 2019; Eschweiler et al., 2022; Moen et al., 2019; Pachitariu and Stringer, 2022; Stringer et al., 2021). In the case of cell-based models of development, microfabrication approaches can be used to constrain cellular organisation and simplify analysis of neighbour relationships (Blin, 2021). One drawback of image analysis-based approaches is that they tend to be limited in the number of markers that can be examined, although advances in multiplexing are rapidly overcoming this limitation, and spatial transcriptomics is rapidly advancing to a stage that it could be routinely applied to complex 3D tissues at single cell resolution (Vandereyken et al., 2023). However, even the most sophisticated spatial transcriptomic assays require cells to be killed before analysis. In contrast, a notable advantage of synthetic neighbour labelling is that it allows identification of neighbours while they are still alive, making it possible to follow their behaviour over time or to extract them and test their properties with functional assays.

Neighbour-profiling methods have huge potential for developmental biology because local short-range interactions govern a wide range of processes in developing tissues. For example, patterning of cell fates can be controlled by local lateral inhibition (Henrique and Schweisguth, 2019) or by reciprocal positive feedback mechanisms such as lateral induction (Petrovic et al., 2014), homeogenetic induction (Linker et al., 2009), quorum-sensing (Chen et al., 2015) or community effects (Addison et al., 2018; Gurdon, 1988; Lowell, 2020; Nemashkalo et al., 2017). Until now, exploring the molecular basis of these events has largely been achieved through genetic or pharmacological screening to identify candidate regulators, which can be laborious and expensive, or by testing the ‘usual suspect’ signalling molecules (e.g. Notch, FGF, Wnt, etc.), which excludes the possibility of identifying unexpected regulators. New methods for unbiased profiling of neighbour responses could provide a new approach to uncover the regulatory logic of cell fate decisions (e.g. does a differentiation event in one cell enhance or repress the same differentiation event in neighbours?), as well as revealing new molecular players that cells use to coordinate cell fate decisions with their local neighbourhood.

Local interactions are also central to ensuring the fitness of developing tissues via cell competition, a process by which healthy cells detect and eliminate defective or ‘unfit’ cells (Bowling et al., 2019; Nichols et al., 2022). Although considerable progress has been made in understanding the cell-autonomous changes that define cellular ‘fitness’, it remains challenging to explore how healthy cells detect unfit neighbours. Similarly, although cells can sometimes sense and respond to differentiation events in their neighbours (Mesa et al., 2018), how this is achieved at the molecular level often remain poorly understood.

In this Primer, we review emerging technologies for enabling neighbour labelling that could help to overcome some of the limitations for studying how cells influence their neighbours during development. We survey synthetic signalling approaches, many of which have been developed for other purposes but which could be applied to developmental questions in future. We also highlight some particularly promising technologies, such as synthetic Notch (synNotch), that have already been used to monitor and manipulate cell behaviours in developmental contexts.

## Synthetic signalling approaches

Synthetic signalling pathways are unnatural cell communication methods, usually generated by combining protein modules that are either artificially designed or co-opted from phylogenetically distant organisms (Chang and Bonnet, 2020; Manhas et al., 2022). Such systems can be engineered in such a way that a given cell type, cell state or cell event triggers the expression of a fluorescent protein in surrounding cells, thus enabling neighbour labelling. For synthetic signalling pathways to be broadly useful for this purpose in studies of development, they must possess the following properties:

(1) Orthogonality to the model system. Synthetic signals should not modulate endogenous signalling pathways, and synthetic receptors should not be modulated by endogenous signals. Furthermore, synthetic signalling pathways should have a minimal impact on cellular resources.
(2) High signal-to-noise ratio. It must be possible to unequivocally distinguish neighbours of cells of interest from non-neighbouring cells.
(3) Lack of cell/tissue type bias. Synthetic neighbour-labelling systems should function with similar efficiency in all cell/tissue types analysed in a study.

**Fig. 1.**
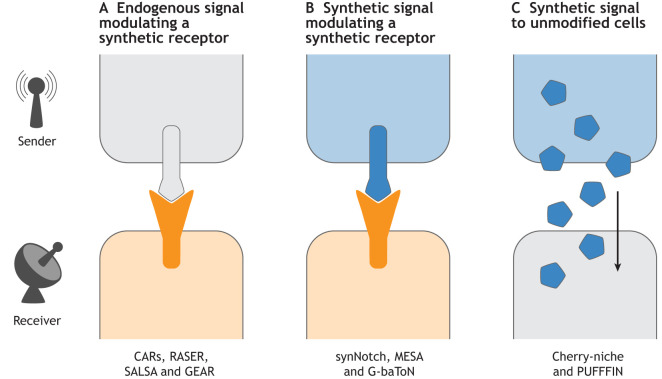
**Synthetic signalling.** Modes of synthetic signalling between a signal-sending (‘sender’) cell and a signal-receiving (‘receiver’) cell. (A) A synthetic receptor in receiver cells is activated by interaction with a natural ligand expressed by unmodified sender cells. Ligands can be membrane tethered or secreted. Examples of this class of synthetic signalling pathways include chimeric antigen receptors (CARs) (Eshhar et al., 1993), rewiring of aberrant signaling to effector release (RASER) (Chung et al., 2019), sensor able to detect lateral signaling activity (SALSA) (Shaffer and Greenwald, 2022) and generalized engineered activation regulator (GEAR) (Krawczyk et al., 2020). (B) A synthetic receptor in receiver cells is activated by its interaction with a synthetic ligand expressed by sender cells. Ligands can be membrane tethered or secreted. Examples of this class of synthetic signalling pathways include synthetic Notch (synNotch) (Morsut et al., 2016), modular extracellular sensor architecture (MESA) (Daringer et al., 2014) and GFP-based touching nexus (G-baToN) (Tang et al., 2020). See main text for other examples. (C) Unmodified receiver cells take up a synthetic secreted molecule expressed in sender cells. Examples of this class of synthetic signalling pathways include Cherry-niche (Ombrato et al., 2019) and positive ultra-bright fluorescent fusion for identifying neighbours (PUFFFIN) (Lebek et al., 2023 preprint). Grey, unmodified; blue, modified sender; orange, modified receiver.

Synthetic signalling pathways can be broadly divided into three classes (Fig. 1): (1) systems relying on an endogenous signal modulating a synthetic receptor; (2) systems relying on a synthetic signal modulating a synthetic receptor; and (3) systems relying on a synthetic signal delivered to unmodified neighbours.

The first class of systems does not possess the orthogonality required for neighbour labelling in complex developmental systems. Recruitment of endogenous signals to synthetic receptors can prove useful to identify neighbours of particular types of signalling cells *in vivo* (Inagaki et al., 2012; Jagadish et al., 2014; Lecourtois and Schweisguth, 1998; Shaffer and Greenwald, 2022; Struhl and Adachi, 1998), but competition for the signal between endogenous and synthetic receptors is likely to impact downstream cellular responses. This Primer, instead, focuses on the second and third class of synthetic signalling pathways because these have the potential to be applied to complex *in vivo* developmental models.

## Systems that rely on a synthetic signal modulating a synthetic receptor

**Fig. 2.**
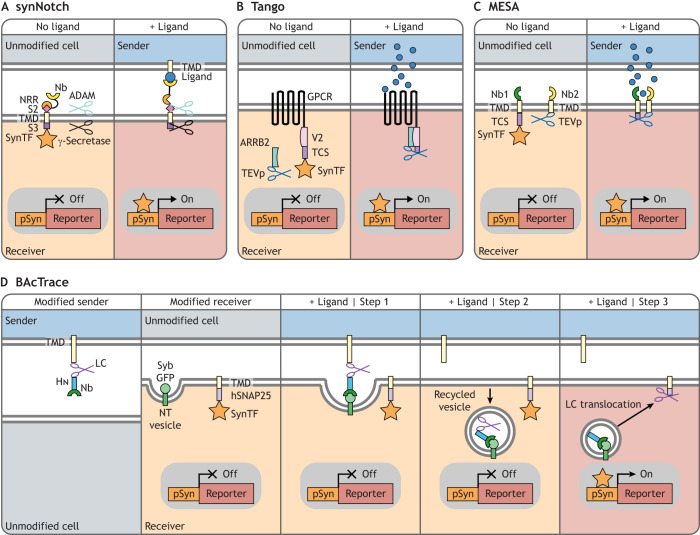
**Class 2 synthetic promoter-based neighbour-labelling systems.** (A) SynNotch: a synthetic re-purposed Notch receptor. The endogenous ligand-binding domain is replaced with a synthetic ligand-binding domain (e.g. a nanobody, Nb) and the intracellular domain is replaced with a synthetic effector, such as a transcription factor (SynTF). After interaction with a membrane-tethered ligand, mechanical tension dislodges the negative regulatory region (NRR) from the S2 cleavage site, allowing two endogenous proteases (ADAM and γ-secretase) to cleave the S2 and S3 sites, releasing the SynTF from the membrane. The SynTF then translocates into the nucleus and activates expression of a reporter transgene from a synthetic promoter (pSyn) (Morsut et al., 2016). TMD, transmembrane domain. (B) Tango: a GPCR is fused to a SynTF via a seven amino acid cleavage site (TCS) recognised by the Tobacco Etch Virus nuclear-inclusion-a endopeptidase (TEVp). TEVp is fused to β-arrestin 2 (ARRB2), a protein that interacts with the V2 peptide of activated GPCRs. Upon binding of a ligand to the Tango GPCR-V2-TCS-SynTF receptor, recruitment of TEVp-ARRB2 leads to cleavage of the TCS, releasing the SynTF from the membrane, which translocates into the nucleus and activates expression of a reporter transgene from a pSyn (Barnea et al., 2008). In the similar system ChaCha (not shown), the GPCR is fused to V2 and TEVp, and ARRB2 is fused to the TCS and an intracellular effector (Kipniss et al., 2017). (C) Modular extracellular sensor architecture (MESA): receiver cells express two receptors that bind different epitopes on the same ligand through different synthetic peptides, such as two nanobodies (Nb1 and Nb2). Upon ligand binding, the two receptors are brought in proximity, allowing the intracellular TEVp on one receptor to cleave the TCS on the other, releasing a SynTF from the membrane, which translocates into the nucleus and activates expression of a reporter transgene from a pSyn (Daringer et al., 2014; Hartfield et al., 2017; Schwarz et al., 2017). (D) Botulinum-activated tracer (BAcTrace): a modified botulinum toxin is fused to the membrane of a sender presynaptic neuron through a TMD. The toxin comprises three domains: LC, a protease that specifically cleaves human SNAP25 (hSNAP25); HN, a translocation domain; and an anti-GFP Nb. Upon interaction with a synaptobrevin-GFP fusion (Syb-GFP) in neurotransmitter (NT) vesicles, the toxin is released from the sender cell and is taken up by receiver neurons after vesicle recycling. The HN domain mediates translocation of LC to the cytoplasm, where it cleaves a membrane-tethered hSNAP25 fused to a SynTF. The SynTF then translocates into the nucleus and activates expression of a reporter transgene from a pSyn (Cachero et al., 2020). In all these systems, sender and/or receiver cells should be labelled with a fluorescent marker (different from the reporter transgene) to easily distinguish sender cells from non-neighbouring receiver cells (shown as cytoplasmic blue or pale orange).

**Fig. 3.**
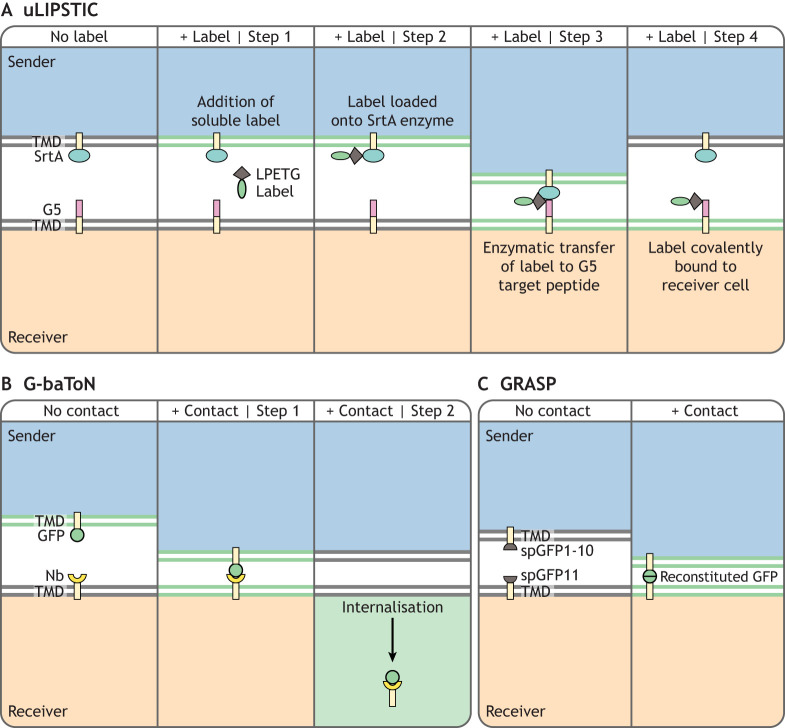
**Class 2 synthetic signal transfer-based neighbour-labelling systems.** (A) Universal labelling immune partnerships by SorTagging intercellular contacts (uLIPSTIC): example of enzymatic proximity labelling of neighbours. An extracellular membrane-tethered bacterial SrtA enzyme on sender cells interacts with a five amino acid substrate (LPETG) that can be bound to a label (e.g. biotin, fluorophore, etc.) at its N terminus. When in proximity of an extracellular membrane-tethered N-terminal pentaglycine peptide (G5) on receiver cells, SrtA catalyses the covalent addition of the label onto the G5 peptide on receiver cells, allowing tracking of historical interactions (Nakandakari-Higa et al., 2023 preprint; Pasqual et al., 2018). TMD, transmembrane domain. (B) GFP-based touching nexus (G-baToN): membrane-tethered extracellular synthetic signal (GFP or mCherry) expressed by sender cells binds to membrane-tethered extracellular anti-GFP or anti-mCherry nanobodies (Nbs) on receivers cells, resulting in fluorophore internalisation by receiver cells (Tang et al., 2020). (C) GFP reconstitution across synaptic partners (GRASP): sender and receiver cells express extracellular membrane-tethered non-fluorescent complementary split GFP fragments (spGFP1-10 and spGFP11). Upon physical interaction, reconstitution of GFP results in fluorescent signal at the junction between the two cells (Feinberg et al., 2008). In all these systems, sender and/or receiver cells should be labelled with a fluorescent marker (different from the reporter transgene) to easily distinguish sender cells from non-neighbouring receiver cells (shown as cytoplasmic blue or pale orange).

In this class of systems, ‘sender’ cells are engineered to express either a secreted or a plasma membrane-tethered synthetic signal, whereas ‘receiver’ cells are engineered to express a synthetic receptor specific to the synthetic signal (Table 1). Signal-receptor interaction leads to labelling of activated receiver cells through a variety of mechanisms, including the expression of fluorescent proteins via a synthetic promoter (Fig. 2) or the transfer of fluorescent molecules from the sender to the receiver cell (Fig. 3).

**Table 1.**
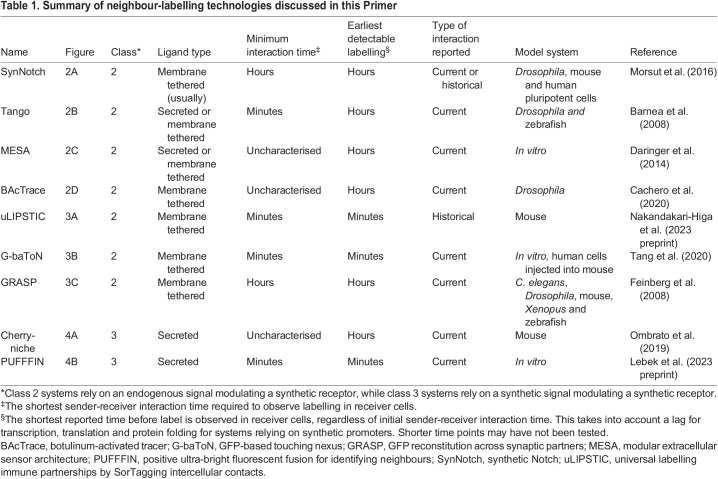
Summary of neighbour-labelling technologies discussed in this Primer

### Synthetic Notch

Synthetic Notch (synNotch) receptors were derived by re-engineering the Notch/Delta signalling cascade (Gordon et al., 2015; Huang et al., 2016; Morsut et al., 2016; Roybal et al., 2016) to produce synthetic responses to synthetic ligands.

Briefly, the extracellular domain of the Notch receptor is engineered to bind to a synthetic ligand (e.g. membrane-tethered GFP on an adjacent cell), while the intracellular domain is replaced by a synthetic transcription factor (e.g. tetracycline transactivator; tTA). Upon ligand binding, mechanical tension exposes a natural Notch cleavage site (Bray, 2016; Gordon et al., 2007), leading to a series of cleavage events (Brou et al., 2000; Mumm et al., 2000; Schroeter et al., 1998; Struhl and Adachi, 2000) that release the intracellular domain, which can then translocate to the nucleus and regulate expression of a transgene (e.g. a fluorescent protein, for neighbour labelling, or a functional protein, for neighbour manipulation) (Fig. 2A) (Gordon et al., 2015; Huang et al., 2016; Morsut et al., 2016; Roybal et al., 2016).

Lim and colleagues performed extensive characterisation of synNotch receptors, illustrating how this system can be used to label and manipulate fate decisions in direct neighbours of mammalian primary, immortalised and cancer cells (Morsut et al., 2016), as well as in primary human T cells (Roybal et al., 2016); this technology has since been implemented in *Drosophila* (Huang et al., 2016) and mouse (Malaguti et al., 2022; Zhang et al., 2022). Applications of synNotch are discussed in more detail below.

### Tango and ChaCha

Tango (Barnea et al., 2008) and a conceptually similar system named ChaCha (Kipniss et al., 2017) were developed to engineer G protein-coupled receptor (GPCR) activation to drive transgene expression (Fig. 2B). Orthogonality to endogenous GPCR signalling has been achieved by using evolved synthetic receptors, which are no longer able to respond to endogenous signals [e.g. the evolved human muscarinic 3 GPCR (hM3D), which recognises the synthetic molecule clozapine-N-oxide (CNO) (Kipniss et al., 2017)], or by using signalling molecules and receptors from distantly related species (e.g. the human glucagon receptor and human glucagon in *Drosophila*) (Sorkaç et al., 2023; Talay et al., 2017). This property has been exploited in the trans-Tango and retro-Tango systems to identify previously unreported cell-cell interactions between gustatory system neurons and specific regions of the *Drosophila* brain (Coomer et al., 2023 preprint; Talay et al., 2017), and to validate previously characterised interactions in the nervous systems of *Drosophila* (Sorkaç et al., 2023; Talay et al., 2017) and zebrafish (Coomer et al., 2023 preprint).

In the trans-Tango system, plasma membrane-bound synapse-localised human glucagon activates a human glucagon-based Tango receptor, labelling post-synaptic neurons adjacent to human glucagon-expressing synapses (Coomer et al., 2023 preprint; Talay et al., 2017). In the retro-Tango system, membrane-bound dendrite-localised human glucagon activates a human glucagon-based Tango receptor, labelling pre-synaptic neurons adjacent to human glucagon-expressing dendrites (Sorkaç et al., 2023).

In both systems, ligands and receptors can be expressed under the control of specific promoters, restricting analysis to interactions between specific cell types (Coomer et al., 2023 preprint; Sorkaç et al., 2023; Talay et al., 2017).

Light-inducible versions of Tango, named SPARK and TRACC, also report interactions in mammalian cell culture models of neural homeostasis and disease, as well as in immortalised cell lines (Cho et al., 2022; Kim et al., 2017, 2019). However, these technologies currently employ endogenous signals to activate synthetic receptors and would, therefore, be unsuitable for whole-organism neighbour labelling.

### MESA

MESA (modular extracellular sensor architecture) receptors rely on two different synthetic receptor molecules that recognise the same synthetic ligand. Ligand binding brings the two receptors into proximity, which in turn triggers the activation of a synthetic response (Fig. 2C) (Daringer et al., 2014; Hartfield et al., 2017; Schwarz et al., 2017).

Although first-generation MESA receptors displayed suboptimal signal-to-noise ratio (Daringer et al., 2014), extensive screening of different receptor architectures by Leonard and colleagues identified signal/receptor combinations with low background reporter expression and robust reporter inducibility (Edelstein et al., 2020). The addition of further layers of control on the receptors, such as degrons and a second TEVp Cleavage Site peptide, also improved signal-to-noise ratio (Zhou et al., 2023). MESA technology is yet to be established in whole organisms, but harbours significant potential for neighbour identification in developmental contexts.

### BAcTrace

The botulinum-activated tracer (BAcTrace) is a retrograde neuronal connection-tracing technology developed in *Drosophila*, based on a modified botulinum toxin that can transfer across synapses and activate a synthetic transcription factor to drive expression of a reporter gene (Fig. 2D). This tool was validated by confirming previous electron microscopy observations on interactomes in the *Drosophila* olfactory system (Cachero et al., 2020).

### Enzymatic proximity labelling

Enzymatic proximity labelling systems make use of enzymes that can transfer a label, such as biotin, to nearby target macromolecules (reviewed by Choi and Rhee, 2022). Originally developed for identifying protein interactomes, enzymes with high target specificity have been exploited for labelling neighbours: sender cells express membrane-tethered labelling enzyme, and receiver cells express membrane-tethered target peptides. The soluble label is enzymatically conjugated to target peptides in the vicinity of sender cells, making these technologies ideal for describing historical interactions.

These systems rely on administration of the labelling substrate to the enzymes, and as such are optimally suited for *in vitro* and *ex vivo* applications. However, they have also been used to identify specific populations of interacting cells *in vivo* in mice and *C. elegans*: LIPSTIC (labelling immune partnerships by SorTagging intercellular contacts) and its derivative system uLIPSTIC (universal LIPSTIC) (Nakandakari-Higa et al., 2023 preprint; Pasqual et al., 2018) (Fig. 3A) were used to identify interactions between T cells and dendritic cells, T cells and B cells, and immune cells and intestinal epithelial cells in mice; iBLINC (*in vivo* biotin labelling of intercellular contacts) (Desbois et al., 2015; Liu et al., 2013) was used to monitor age-dependent changes in synaptic connections between AFD and AIY interneurons in *C. elegans*.

Other systems used to study cell interactions *in vitro* include ID-PRIME (interaction-dependent probe incorporation mediated by enzymes) (Liu et al., 2013), PUP-IT (pupylation-based interaction tagging) (Liu et al., 2018) and TransitID (trafficking analysis by sequentially incorporated tags for identification) (Qin et al., 2023).

For several of these systems, label-loaded sender cells that have not yet interacted with receiver cells cannot be distinguished from labelled receiver cells on the basis of label alone. Expression of ligand and receptor in different cell types allows separation of senders and receivers based on expression of markers of cell identity (Nakandakari-Higa et al., 2023 preprint; Pasqual et al., 2018). Alternatively, sender and/or receiver cells can be engineered to express an intracellular fluorescent protein to aid in their identification (Liu et al., 2018).

### G-baToN

GFP-based touching nexus (G-baToN) is a protease- and effector-independent direct neighbour-labelling method that relies on a membrane-bound synthetic signal (GFP or mCherry) being transferred to neighbouring cells containing membrane-tethered anti-GFP or anti-mCherry nanobodies (Fig. 3B). The system works in a wide range of mammalian immortalised, cancer and primary cells, and can detect interactions between human T cells and tumours in mice (Tang et al., 2020).

### GRASP

GRASP (GFP reconstitution across synaptic partners) is a technology that allows users to identify cells in contact through imaging. It relies on the reconstitution of extracellular membrane-tethered non-fluorescent split GFP fragments into a functional GFP molecule by neurons in close proximity (Fig. 3C), and has been used to map neural connections in *C. elegans*, *Drosophila* and mice (Feinberg et al., 2008; Gordon and Scott, 2009; Kim et al., 2012; Yamagata and Sanes, 2012). The requirement for interaction between split GFP fragments ensures that fluorescence reports only current cell-cell interactions, and allows only imaging-based neighbour-identification in intact tissues, and not isolation of cells by flow cytometry.

A conceptually similar technology named GRAPHIC (glycophosphatidylinositol anchored reconstitution-activated proteins highlight intercellular connections) was used to identify cell interactions in the mouse brain, zebrafish retina, *Xenopus* neural tube and cultured rat hippocampal neurons, as well as between non-neural cultured cells (Kinoshita et al., 2019, 2020).

## Systems relying on a synthetic signal delivered to unmodified neighbours

The approaches described above require engineering of sender and receiver populations. Simpler systems for neighbour labelling do not require any genetic modification of receiver cells, and can thus be applied more readily to developmental systems without the need to generate transgenic animal lines (Table 1). For example, engineered sender cells could be incorporated into embryos by grafting, transplantation or the construction of chimeric animals (Tam and Rossant, 2003; Stern, 2005; Huang et al, 2015). Alternatively, ‘sender’ plasmids can be delivered directly into cells of interest within model organisms (Nakamura et al., 2004; Huang et al, 2015). There are so far very few examples of systems that are able to label unmodified neighbours; we discuss these in detail below.

### Cherry-niche

**Fig. 4.**
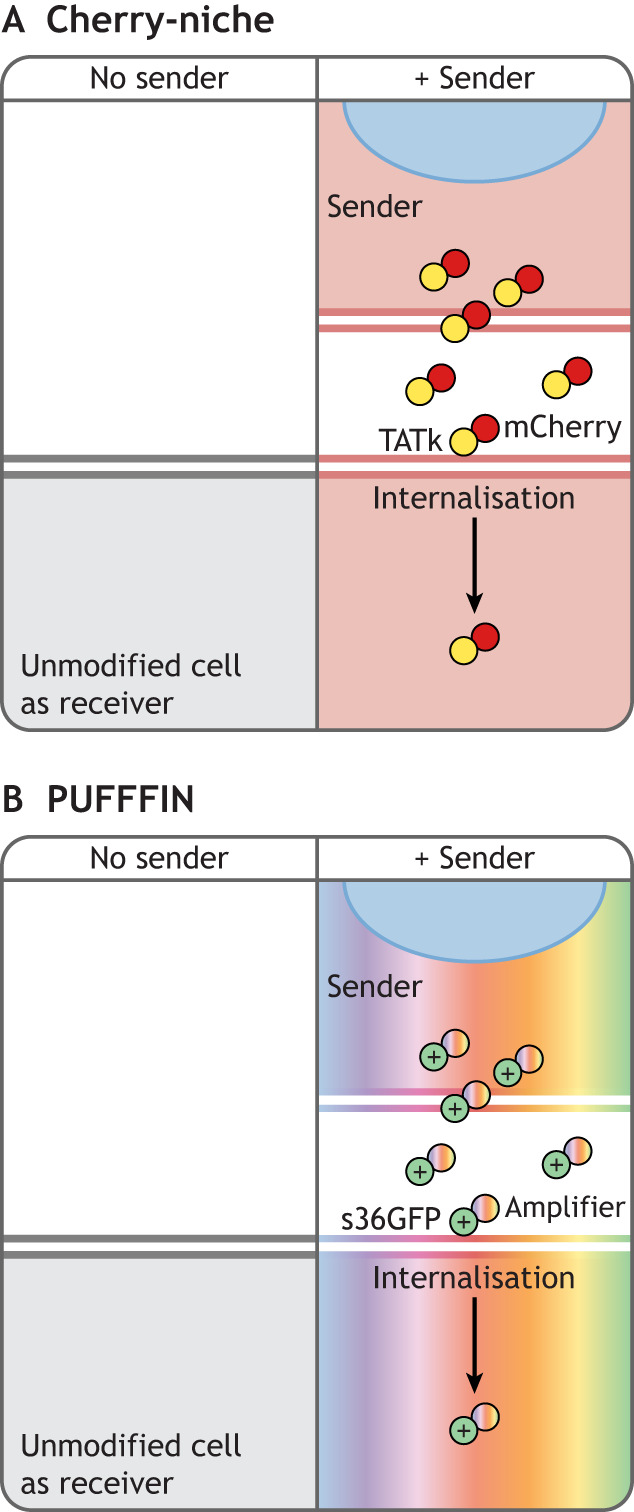
**Class 3 neighbour-labelling systems: synthetic signal delivered to unmodified neighbours.** (A) Cherry-niche: mCherry is fused to a signal peptide, driving its secretion from sender cells, and a TATk cell-penetrating peptide, which mediates its uptake by unmodified neighbours (Ombrato et al., 2019). (B) Positive ultra-bright fluorescent fusion for identifying neighbours (PUFFFIN): s36GFP (a highly positively charged GFP fused to a signal peptide to drive its secretion from sender cells) is fused to an amplifier peptide (e.g. ultra-bright fluorescent protein, HaloTag). The high positive charge of s36GFP mediates its interaction with negatively charged plasma membranes of neighbouring cells, resulting in its uptake by unmodified neighbours. Use of HaloTag as an amplifier allows labelling of neighbours with HaloTag ligands of any colour (Lebek et al., 2023 preprint). In both these systems, sender cells should be labelled with a fluorescent marker (different from the secreted signal; shown as a blue nucleus) to distinguish sender cells easily from labelled neighbouring receiver cells.

The Cherry-niche system was developed to study cell interactions in the tumour microenvironment (Ombrato et al., 2019; Ombrato et al., 2021). The monomeric fluorescent protein mCherry was engineered with an N-terminal signal peptide to enable it to be secreted from sender cells using endogenous secretory machinery (Barash et al., 2002), together with a liposoluble Transactivator of Transcription (TATk) domain that enables fluorescent protein uptake by unmodified neighbouring cells (Flinterman et al., 2009) (Fig. 4A). 4T1 breast cancer cells were engineered to express this cell-penetrating mCherry protein and a cytoplasmic GFP, to enable mCherry^+^ GFP^+^ ‘sender’ cells to be distinguished from any mCherry^+^ GFP^−^ labelled neighbours. These neighbour-labelling cells were injected into mice where they formed tumours. Labelled neighbours were then extracted and profiled to characterise the tumour microenvironment (Nolan et al., 2022; Ombrato et al., 2019). A Cre-inducible version of Cherry-niche has also been used to study homeostatic interactions between hepatocytes and endothelial cells in adult mice (Zhang et al., 2023)

### PUFFFIN

A technology called PUFFFIN (positive ultra-bright fluorescent fusion for identifying neighbours) (Lebek et al., 2023 preprint) was designed with developmental applications in mind. This system exploits the ability of a highly positively charged GFP (+36GFP) to cross cell membranes (Cronican et al., 2010; McNaughton et al., 2009; Thompson et al., 2012). The addition of a signal peptide allows its secretion by sender cells, and fusion with a fluorescence amplifier enables ultra-bright labelling that becomes detectable in unmodified neighbours in under 1 h (Lebek et al., 2023 preprint). The signal amplifier can be either an ultra-bright fluorescent protein or a HaloTag (a non-fluorescent protein tag that covalently binds and activates cell-permeable fluorogenic dyes) (Los et al., 2008). This approach enables colour-of-choice fluorescent labelling, without the need for additional genetic manipulation, making the system readily compatible with any existing fluorescent reporter cell line (Lebek et al., 2023 preprint) (Fig. 4B). It also uses a modular customisable design to allow expression of the label from any promoter (e.g. an inducible or cell-type-specific driver) or co-expression of the label with a transgene of interest (e.g. a pro-differentiation gene), all within a single plasmid (Lebek et al., 2023 preprint). This offers a simple and flexible approach to manipulate differentiation (or other cell properties) in one cell while at the same time labelling its neighbours.

### Other approaches

Other approaches for transferring labelling molecules between cells have been developed for various purposes. For example, viral transneuronal tracing has been used for many years to map neuronal connections in rodents and *Xenopus* (Ugolini et al, 1989; Ugolini, 1995; Faulkner et al., 2021), allowing isolation and omics profiling of neurons projecting into specific areas of the mouse brain (Kim et al., 2020; Zhang et al., 2021) and the identification of neighbours of individual infected sender neurons in rat brain slice cultures (Wickersham et al., 2007). However, infection with tracer virus may affect cellular behaviour (Patiño et al., 2022).

Inter-cellular transfer of fluorescein-labelled farnesylated chemically self-assembled nanorings (f-CSAN) has been used to deliver cargo to neighbours of primary and cancer sender cells (Wang et al., 2022), but f-CSANs need to be bound to sender cells before use, limiting its applicability to *in vitro* or grafting experiments, even if established in developmentally relevant models.

Several cell penetrating peptides have been described (Gagat et al., 2017). These could facilitate label transfer to neighbouring cells in a manner similar to the Cherry-niche and PUFFFIN if established *in vivo* or in cell culture models of development.

Having access to a more diverse range of neighbour-labelling systems would prove particularly useful should these technologies exhibit differences in the dynamics or range of labelling: they could be used to profile different types of neighbourhoods (direct neighbours versus nearby cells) and interactions (transient versus sustained), and/or could be combined to study neighbour responses to two or more different events within a single experimental system.

## Opportunities and limitations of synNotch for developmental studies

Of the synthetic signal/synthetic receptor systems, synNotch seems particularly promising for neighbour labelling during development. This is exemplified by work carried out in *Drosophila*, where synNotch was used not only to validate cellular interactions previously reported through image analysis, such as those between olfactory receptor neurons and projection neurons, but also to identify previously uncharacterised interacting partners, such as tracheal cells and myoblasts (He et al., 2017; Huang et al., 2016, 2017). Lois and colleagues (Huang et al., 2016, 2017) were also able to describe a previously unreported subset of glia based exclusively on their interaction with olfactory neurons, illustrating how synNotch can be used to define cell populations in the absence of population-defining markers.

SynNotch has been fine-tuned for reliable and efficient neighbour labelling in mouse pluripotent cells and chimaeric embryos, through a series of optimisations to minimise false positive (labelled non-neighbours) and false negative (unlabelled neighbours) labelling. This system, termed SyNPL, benefits from a modular cassette exchange design that allowed the authors to conveniently replace the fluorescent label transgene with a pro-differentiation transgene. This resulted in contact-mediated synthetic patterning of two distinct cell types (a stripe of neurons across a field of pluripotent cells) without the need for an exogenous differentiation regime (Malaguti et al., 2022). In a particularly impressive feat of bioengineering, synNotch has been used to generate Crispr-engineered mouse lines to either monitor cell interactions in real time or to track the history of interactions over developmental time, using cell contact to induce Cre recombinase (rather than a fluorescent label) and combining this with Cre-dependent lineage labelling (Liu et al., 2023; Zhang et al., 2022) (see also below).

Human pluripotent synNotch receiver cells have been engineered to express a fluorescent label and a functional transgene in response to a synthetic ligand-patterned surface. This system has been used to accelerate neuronal induction in a neural differentiation regime (Lee et al., 2023). It is likely that the label would also be induced by ligand-presenting sender cells, although this remains to be tested.

**Fig. 5.**
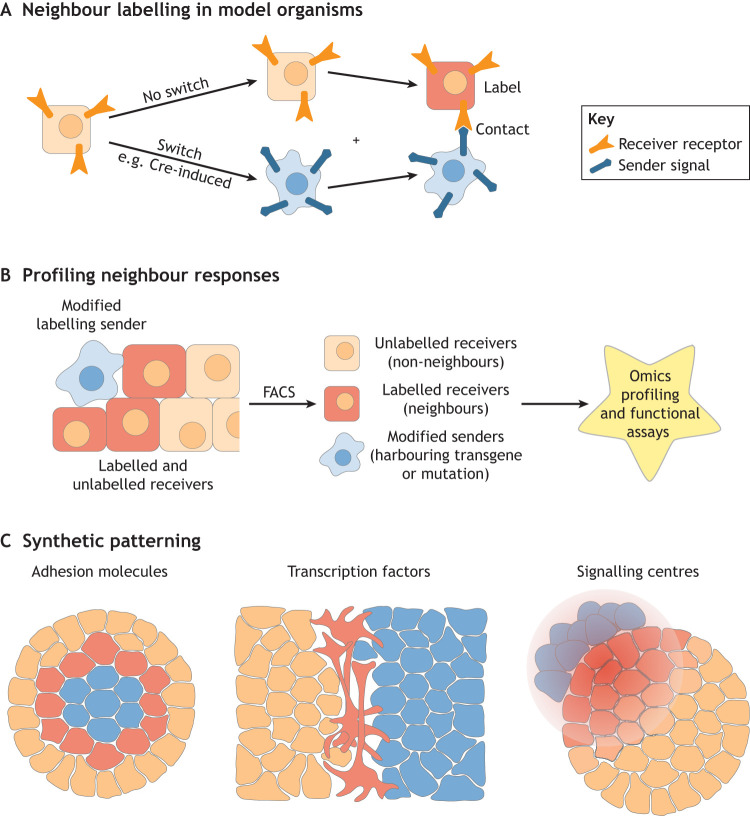
**Applications of neighbour labelling in developmental systems.** (A) An adaptation of synthetic Notch (synNotch) technology that uses a recombinase to convert receiver cells into sender cells. This technology has been applied in *Drosophila* (He et al., 2017) and mouse (Zhang et al., 2022). (B) Neighbour-labelling technologies offer the opportunity to use FACS to sort neighbours of a particular cell type and use omics approaches to identify differences between these neighbours and more-distant cells within a tissue, or to carry out functional assays on the live isolated neighbours and non-neighbours. (C) Synthetic signalling technologies can also be applied to engineer synthetic patterning. (Left) synNotch can be used to propagate changes in adhesion to programme self-organisation (Toda et al., 2020); (Middle) synNotch can drive a pro-neural transcription factor at the boundary between senders and receivers to generate a stripe of neurons within a dish of embryonic stem cells (Malaguti et al., 2022; see also Garibyan et al., 2023 preprint; Gordon et al., 2015; Lee et al., 2023; Morsut et al., 2016). (Right) Illustration of the use of artificial signalling centres to synthetically pattern groups of cells (Cederquist et al., 2019; Davies, 2022; Glykofrydis et al., 2021; Manfrin et al., 2019).

A key property of synNotch technology is that synNotch receptors are inhibited by ligands expressed in *cis* (i.e. on the same cell as the receptor) (Huang et al., 2016; Morsut et al., 2016). This implies that either the expression of receptor and ligand constructs has to be regulated by mutually exclusive tissue-specific promoters, or that this technology has to be used in chimaeric systems, comprising separate ‘sender’ and ‘receiver’ cells that express ligand and receptor, respectively. This could, in principle, limit applicability in developmental model organisms. To overcome this problem, Perrimon and colleagues devised a *Drosophila* line in which the synNotch receptor is flanked by tandem FRT site-specific recombination sites, followed by a synNotch ligand, initially not expressed. This results in a fly in which all cells are initially ‘receiver’ cells. Upon expression of Flp recombinase by a lineage-specific and/or inducible promoter, the synNotch receptor is excised from the genome through Flp/FRT recombination, and the synNotch ligand is expressed in its place, converting Flp-expressing cells into ‘sender’ cells, which in turn label their ‘receiver’ neighbours (He et al., 2017). A similar strategy, involving Cre/loxP recombination in place of Flp/FRT recombination, was adopted by Zhou and colleagues in mouse embryos (Liu et al., 2023; Zhang et al., 2022). By crossing these organisms with promoter-specific Flp/Cre-driver lines, these ‘all-in-one’ systems offer great modularity for identifying neighbours of any cell type of interest, without the need for recurrent genetic targeting of synNotch receptors and ligand to different promoters to avoid *cis*-inhibition (Fig. 5A).

**Fig. 6.**
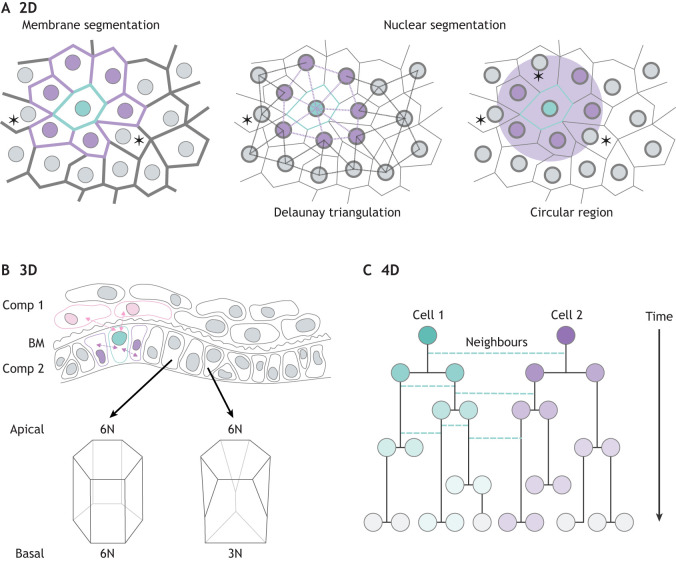
**Defining neighbours.** Defining neighbours within images seems conceptually simple but can be technically challenging. (A) In 2D images with membrane segmentation, neighbours can be identified based on direct cell contact. However, decisions must be made about the extent of contact required to justify neighbour status (e.g. cells labelled with an asterisk). Nuclear segmentation in the absence of cell membrane segmentation creates uncertainty about whether any two given nuclei reside within two cells that are directly in contact or whether their host cells are separated by the cytoplasm of an intervening cell. To help overcome this problem, cell neighbours can be approximated using approaches such as Delaunay triangulation or by making assumptions based on measuring nearest-neighbour nuclei within a defined threshold distance. Turquoise, cell of interest; lilac, neighbours identified by each method. (B) 3D images can present additional uncertainty in defining neighbours, particularly when cells have irregular shapes or when two different cellular compartments (labelled as comp 1 and comp 2) are separated by a basement membrane (BM) (N, number of neighbours). For example, are the pink cells neighbours of the turquoise cell, or are the lilac cells the only neighbours of the turquoise cell? (C) Further problems arise when analysing neighbour relationships over time because of the complexity of generating useful lineage trees based on non-cell-autonomous cell relationships.

A simultaneous strength and limitation of the standard form of synNotch technology is the requirement for direct contact between a membrane-tethered ligand and the synNotch receptor. This ensures all cells labelled by a ligand-expressing ‘sender’ cell are its true neighbours, but it also makes it impossible to distinguish between unlabelled cells that are close to or far from the sender cell. However, a variation of synNotch technology was developed to label non-direct neighbours in signalling range. Toda, Lim and colleagues engineered three types of immortalised mouse fibroblasts: ‘secretor’ cells secreting a soluble synthetic signal; ‘anchor’ cells carrying a membrane-tethered high-affinity nanobody capable of recognising the soluble signal; and ‘receiver’ cells carrying synNotch receptors with a low-affinity nanobody against the soluble signal. The anchor cells bind the diffusible secreted signal and act as ‘sender’ cells, generating sufficient mechanical tension upon their interaction with the synNotch receptor on receiver cells to drive receptor cleavage (Toda et al., 2020).

Other variations on synNotch technology include modified receptors that require higher mechanical tension to drive receptor cleavage (Sloas et al., 2023); receptors with optimised extracellular, transmembrane or juxtamembrane domains (Yang et al., 2020; Zhu et al., 2022); and receptors that are activated in *cis* rather than in *trans* (Mahameed et al., 2022). There is also evidence that synNotch receptors can be activated by extracellular matrix-bound ligands (Garibyan et al., 2023 preprint; Gordon et al., 2015; Lee et al., 2023; Morsut et al., 2016). These examples of synNotch re-engineering illustrate the versatility of this technology for identifying the cellular neighbourhood in development.

Despite its many advantages, even the powerful and flexible synNotch system requires considerable genome engineering, and applying it *in vivo* requires the generation of transgenic animals (He et al., 2017; Huang et al., 2016, 2017; Liu et al., 2023; Zhang et al., 2022). In contrast, Cherry-niche and PUFFFIN systems require only the delivery of plasmids (in the case of PUFFFIN, only a single plasmid) to sender cells, with no need to engineer receiver cells. This could become particularly useful, for example, in experiments where labelled senders can be grafted or transplanted into wild-type embryos, tissues or organoids, or where the label could be delivered by electroporation to a defined ‘sender’ population within a wild-type embryo. These approaches therefore offer broad applicability to studies of development.

## Other considerations when using neighbour-labelling approaches for developmental studies

When establishing neighbour-labelling models, it is important to ensure that sender and receiver cells can be easily distinguished. For class 2 systems relying on transcriptional activation of a reporter in receiver cells (e.g. synNotch, Tango, MESA and BAcTrace), a sender cell is indistinguishable from a non-neighbouring receiver (which is not transcribing the reporter transgene) on the basis of reporter gene expression alone (Cachero et al., 2020; Daringer et al., 2014; Morsut et al., 2016; Talay et al., 2017). For class 2 and class 3 systems relying on transfer of a fluorescent label (uLIPSTIC, G-baToN, Cherry-niche and PUFFFIN), a label-secreting sender cell is indistinguishable from a labelled receiver neighbour on the basis of label alone (Lebek et al., 2023 preprint; Nakandakari-Higa et al., 2023 preprint; Ombrato et al., 2019; Tang et al., 2020). It is, therefore, crucial to ensure that sender and/or receiver cells express a distinct intracellular fluorescent marker, to allow unequivocal identification of cells as senders and/or receivers (Malaguti et al., 2022).

Different neighbour-labelling systems differ in the duration of contact needed to transfer or induce neighbour labelling (Table 1). For example, the SyNPL system requires a minimum of 2 h contact between senders and receivers (Malaguti et al., 2022), which is an advantage for experiments that wish to focus only on the outcome of relatively stable cell-cell interactions. In contrast, the PUFFFIN system can transfer label in under 1 h (Lebek et al., 2023 preprint), making it useful for studies of both transient and stable cell interactions. In both cases, the fluorescent label persists over many hours, making it possible to record ‘historical’ as well as current interactions, although of course it should be possible in principle to adjust this time frame by switching to destabilised versions of the labelling proteins.

It is important to bear in mind that the activity of each system may be cell-type dependent. For example, it has been speculated that the Cherry-niche system may be most useful in cell types that are highly secretory, such as cancer cells (Ombrato et al., 2019; Ombrato et al., 2021). Similarly, synNotch activity may vary between cell types according to the expression of the appropriate Notch-cleavage machinery. It would, therefore, be prudent to assess the activity of the chosen neighbour-labelling system within all cell types likely to be represented within a given study.

Class 2 synthetic promoter-based neighbour-labelling systems (e.g. synNotch, Tango, MESA) are well suited to neighbour labelling for unbiased profiling of neighbour interactions (Fig. 5B), but perhaps their greatest strength is that they can be adapted to drive any transgene of interest in receiver cells in response to contact with sender cells. This makes it possible to use these tools to engineer synthetic patterning of cell fate or tissue morphology (Fig. 5C) (Davies, 2017; Trentesaux et al., 2023).

As a note of caution, both the synNotch study performed by Huang et al. (2017) and the trans-Tango study performed by Talay et al.(2017) sought to identify neighbours of olfactory neurons in *Drosophila*, with the two technologies revealing different numbers of neighbours. This serves as a reminder that the exciting potential of neighbour identification via synthetic signalling systems is still in its infancy, and should be carefully validated. For example, if a particular neighbour response is identified using synthetic neighbour labelling, then image analysis-based approaches (Box 1; Fig. 6) could be used to validate this response within intact tissues.

## Conclusion

There has been great progress made over recent decades in understanding how cell-cell interactions govern development, but until recently these studies have often relied on low-throughput testing of candidate signals, resource-heavy screening strategies (St Johnston, 2002) and painstaking direct observation over time (White et al., 1986). The approaches summarised in this Primer provide a toolkit for relatively straightforward unbiased discovery of the mechanisms that cells use to communicate with each other to orchestrate the magnificent process of embryonic development.
